# Innate immune response in bovine neutrophils stimulated with *Mycoplasma bovis*

**DOI:** 10.1186/s13567-021-00920-2

**Published:** 2021-04-16

**Authors:** Satoshi Gondaira, Koji Nishi, Jumpei Fujiki, Hidetomo Iwano, Reina Watanabe, Ayako Eguchi, Yuki Hirano, Hidetoshi Higuchi, Hajime Nagahata

**Affiliations:** 1 Animal Health Laboratory, Department of Veterinary Medicine, School of Veterinary Medicine, Rakuno Gakuen University, Ebetsu, Hokkaido 069-8501 Japan; 2 Veterinary Biochemistry, Department of Veterinary Medicine, School of Veterinary Medicine, Rakuno Gakuen University, Ebetsu, Hokkaido 069-8501 Japan; 3 Animal Research Center, Agricultural Research Department, Hokkaido Research Organization, Shintoku, Hokkaido 081-0038 Japan; 4 Farm Animal Veterinary Nursing Laboratory, Department of Veterinary Associated Science, Faculty of Veterinary Medicine, Okayama University, Imabari, Ehime 794-8555 Japan

**Keywords:** *Mycoplasma bovis*, Bovine, Neutrophils, Innate immune, Transcriptome analysis

## Abstract

*Mycoplasma bovis* (*M. bovis*) is a significant worldwide pathogen of cattle. Neutrophils have an important role in the innate immune response during infection with *M. bovis*. However, even though neutrophils accumulate in *M. bovis* infection, the interaction of *M. bovis* and neutrophils has not been fully elucidated. We attempted to elucidate the innate immune response of neutrophils stimulated with *M. bovis* and evaluate the transcriptome and functional analysis of bovine neutrophils stimulated with *M. bovis*. Proinflammatory cytokines, such as inducible nitric oxide (*iNOS*), which was the most increased gene in transcriptome analysis, were increased in quantitative polymerase chain reaction analysis of bovine neutrophils stimulated with live or heat-killed *M. bovis*. Nitric oxide and intracellular reactive oxygen species production of neutrophils stimulated with *M. bovis* was significantly increased. Neutrophils stimulated with *M. bovis* showed an increased ratio of nonapoptotic cell death compared to unstimulated controls. We demonstrated that neutrophil extracellular traps (NETs) formation was not recognized in neutrophils stimulated with live *M. bovis*. However, heat-killed *M. bovis* induced NETs formation. We also showed the interaction with *M. bovis* and bovine neutrophils regarding proinflammatory cytokine gene expression and functional expression related to NETs formation. Live and killed *M. bovis* induced innate immune responses in neutrophils and had the potential to induce NETs formation, but live *M. bovis* escaped NETs.

**Supplementary Information:**

The online version contains supplementary material available at 10.1186/s13567-021-00920-2.

## Introduction

Mycoplasmas are classified under the class Mollicutes, which do not have a cell wall and are cause widespread infections of eukaryotes in nature [1]. *Mycoplasma bovis* (*M. bovis*) is a significant worldwide pathogen of cattle [2, 3] and is known to cause pneumonia, arthritis, and mastitis [2, 4], resulting in calf mortality, weight loss in surviving calves, and decreased milk production in dairy cows [2, 5], which all contribute to significant economic losses [2, 6].

Neutrophils have an important role in infectious diseases at the front line. In *M. bovis* infection, neutrophils constitute the major accumulation of cells at an infection site [7]. *M. bovis* reportedly suppressed the production of reactive oxygen species (ROS) in the immune response of neutrophils [8]. ROS is the major innate immune factor of neutrophils to pathogens and is required for neutrophil extracellular traps (NETs) formation [9]. ROS and inducible nitric oxide (*iNOS*) are involved in the pathogenesis of *Mycoplasma* pneumoniae calves [10], and nitric oxide (NO) also triggers and enhances the release of NETs [11]. *M. bovis* was considered to escape the host immune response, and we previously reported that *M. bovis* escaped bovine NETs following the degradation of nucleic acid [12].

Inflammatory cytokines have an important role in the innate immune responses of neutrophils, such as recruitment, activation, and NETs formation [13]. The suppression of NO production and increased gene expression of inflammatory cytokines, such as IL-12 and TNF-α, as a response of neutrophils to *M. bovis*, have been previously reported [14]. However, the mechanisms of the neutrophil immune response involved in *M. bovis* comprehensive gene expression have not been fully elucidated. We attempted to elucidate the innate immune response of neutrophils stimulated with *M. bovis*, determining gene expression related to the innate immune response through comprehensive gene expression analysis, and to determine whether *M. bovis* is capable of inducing NETs formation.

## Materials and methods

### Bacterial strains

The bacterial strain used in this study was *M. bovis* (PG45: ATCC 25,523), grown in modified pleuropneumonia-like organisms (PPLO) medium (Kanto Kagaku, Tokyo, Japan) at 37 °C for 48 h. *M. bovis* was obtained by centrifugation (16 000 *g* for 40 min) and then washed with phosphate-buffered saline (PBS). The bacteria were then suspended in PBS to a cell density of 10^8^ colony-forming units per milliliter (CFU/mL), and the suspension was stored at −70 °C until used.

### Bovine neutrophils

Blood samples (20 mL) were collected in evacuation tubes containing sodium heparin (Terumo, Tokyo, Japan) from eleven clinically healthy primiparous Holstein cows in mid-lactation with no history of *M. bovis* infection. Three cows were used for microarray analysis and five cows were used for validation experiments using real-time PCR, detection of apoptotic cells, quantity of NO, and ROS production. Another three cows were used for the observation of NETs formation. The experimental protocol was approved by the Institutional Animal Care and Use Committee of Rakuno Gakuen University. Neutrophils were isolated by centrifugation on a Lympholyte device (Cedarlane, Ontario, Canada) according to the manufacturer’s protocol. Cells were separated by centrifugation (300 *g* for 30 min), and neutrophils were transferred to a sterile tube (Becton Dickinson, Tokyo, Japan) and washed with cold PBS. Neutrophil viability was assessed using an AO/PI cell viability kit (Logos Biosystems, Gyeonggi, Korea) and Luna-FL (Logos Biosystems). Neutrophil ratios in polymorphonuclear leukocytes (PMNL) were obtained following Diff-Quick staining (Sysmex, Hyogo, Japan). Neutrophils were suspended in Hank’s balanced salt solution or RPMI 1640 medium with L-glutamine (Sigma-Aldrich Corp., Tokyo, Japan) and 10% fetal bovine serum (FBS). Isolated neutrophils from five individual cows were used immediately. Neutrophils (concentration of 1 × 10^7^ cells in 3 mL RPMI 1640 medium) were incubated in the presence of live or heat-killed *M. bovis* at a multiplicity of infection (MOI) of 1000:1 for 3 and 6 h at 37 °C and 5% CO_2_ in 60-mm dishes (Asahi Glass, Tokyo, Japan). The number of bacteria in the milk of *M. bovis* mastitis was of 10^9^ to 10^11^ CFU and the number of somatic cell counts was 10^6^ to 10^7^ cells/mL [15]. Thus, MOI of 1000 is a sufficiently reasonable number of bacteria, taking into account the actual infection.

### RNA extraction

Total RNA (TRNA) extracted from neutrophils was obtained using the PureLink RNA mini kit (Ambion, TX, USA). DNAse digestion was performed using TURBO DNA-free DNAse (Ambion). TRNA was quantified via spectrophotometry using a BioSpec-nano (Shimadzu, Kyoto, Japan). cDNA was synthesized from 1 μg TRNA with ReverTra Ace reverse transcriptase (Toyobo, Osaka, Japan) and oligo dT primers (Toyobo). For each reaction, a parallel negative control reaction was performed in the absence of reverse transcriptase and analyzed via the β-actin band using polymerase chain reaction (PCR) and 1.5% agarose gels stained with ethidium bromide and visualized on an ultraviolet transilluminator.

### Microarray experiment and analysis

Six microarray (three stimuli and three control) data for the neutrophils stimulated with *M. bovis* for 3 h were provided by Takara Bio, Inc. (Siga, Japan). The gene expression dataset was obtained using an Agilent single-color microarray platform (4 × 44 K bovine gene expression array, grid ID 023,647). Samples were processed for Agilent microarrays, and data were normalized as described previously [16]. We used *t*-tests to identify significant gene expression differences (*P* < 0.025) between samples. In a further filtering step, we selected only genes with a fold change of ≥ 2. The gene annotation used was bioDBnet [17]. Heat map analysis was done using R version 3.6.1, and gene ontology enrichment was done using the BioMart enrichment tool [18]. The whole dataset is available publicly from the ArrayExpress database (accession number E-MTAB-9022).

### Quantitative reverse transcription PCR (qPCR) analysis

The reaction was performed using a Thunderbird SYBR qPCR mix (Toyobo) and a CFX real-time PCR system (Bio-Rad Laboratories, Hercules, CA, USA). Information on the primers is depicted in Additional file 1. We used the melting curve analysis to evaluate each primer pair for specificity to ascertain that only one product was amplified. We performed a Basic Logical Alignment Search Tool (BLAST) search to confirm that the primer sequences amplified only the target gene of interest. Thermal cycling consisted of initial denaturation at 95 °C for 5 min, followed by 40 cycles of denaturation at 95 °C for 15 s, annealing at 60 °C for 30 s, and extension at 72 °C for 30 s. The melting temperature of the PCR product was determined by melting curve analysis, which was performed by heating the PCR product from 55 °C to 95 °C and monitoring the fluorescence change every 0.5 °C. β-actin, glyceraldehyde-3-phosphate dehydrogenase (*GAPDH*), and tryptophan 5-monooxygenase activation protein zeta polypeptide (*YWHAZ*) were used as reference genes [19, 20].

### Detection of apoptotic cells and quantity of NO and ROS production

To evaluate NO production and the ratio of apoptosis cells in neutrophils stimulated with *M. bovis*, neutrophils (2 × 10^5^ cells/200 μL RPMI 1640 medium in 96-well tissue culture plates) were incubated in the presence of live *M. bovis* at an MOI of 1000 (10 μL) for 1, 3, and 6 h at 37 °C and under 5% CO_2_. The NO production and ratio of apoptosis cell were measured using a Muse NO kit or Muse Annexin V and dead cell kit (Millipore, Darmstadt, Germany) and Muse cell analyzer (Millipore) according to the manufacturer’s protocol. To measure the quantity of intracellular ROS in neutrophils stimulated with *M. bovis*, neutrophils (2 × 10^5^ cells/1 mL RPMI 1640 medium in a 3 cm dish) were stimulated with 10 μL conteining live *M. bovis* and/or phorbol myristate acetate (PMA; Sigma-Aldrich Corp.) or PBS for control at an MOI of 1000 for 30 min at 37 °C and 5% CO_2_. After that, intracellular ROS production was detected using the Muse Oxidative stress kit and Muse cell analyzer (Millipore) according to the manufacturer’s protocol.

### Observation of NETs formation

Neutrophils (concentration of 1 × 10^6^ cells suspended in 100 μL RPMI medium with 10% FBS) were seeded onto glass coverslips treated with 0.001% poly-l-lysine (Matsunami glass, Tokyo, Japan) and placed in a 35 mm dish (Iwaki, Shizuoka, Japan). Cells were incubated for 1 h at 37 °C in 5% CO_2_. Neutrophils were incubated with PMA for 30 min to induce NETs formation (or with PBS for control), and then, 10^7^ CFU octadecyl rhodamine B chloride (Sigma-Aldrich Corp.) labeled live or heat-killed *M. bovis* (or with PBS for control) were added and incubated for 30 min at 37 °C under a 5% CO_2_. Neutrophils were washed with PBS and stained with 4,6-diamidino-2-phenylindole, dilactate (DAPI) for 15 min (Dojindo, Tokyo, Japan). Coverslips were washed with PBS, coated with Fluoromount (Diagnostic Biosystems, Pleasanton, CA, USA), and viewed using a fluorescence microscope (Nikon, Tokyo, Japan). Three bovine neutrophil studies were performed individually.

### Statistical analysis

Data from five cows were expressed as mean ± standard error (SE). The Kruskal–Wallis test was performed for comparison between groups, Steel test for multiple comparisons, and Welch’s *t*-test for paired groups using Ekuseru-Toukei 2010 for Windows (Social Survey Research Information, Tokyo, Japan). In all cases, *P* < 0.05 was considered statistically significant.

## Results

### Microarray analysis

**Figure 1:**
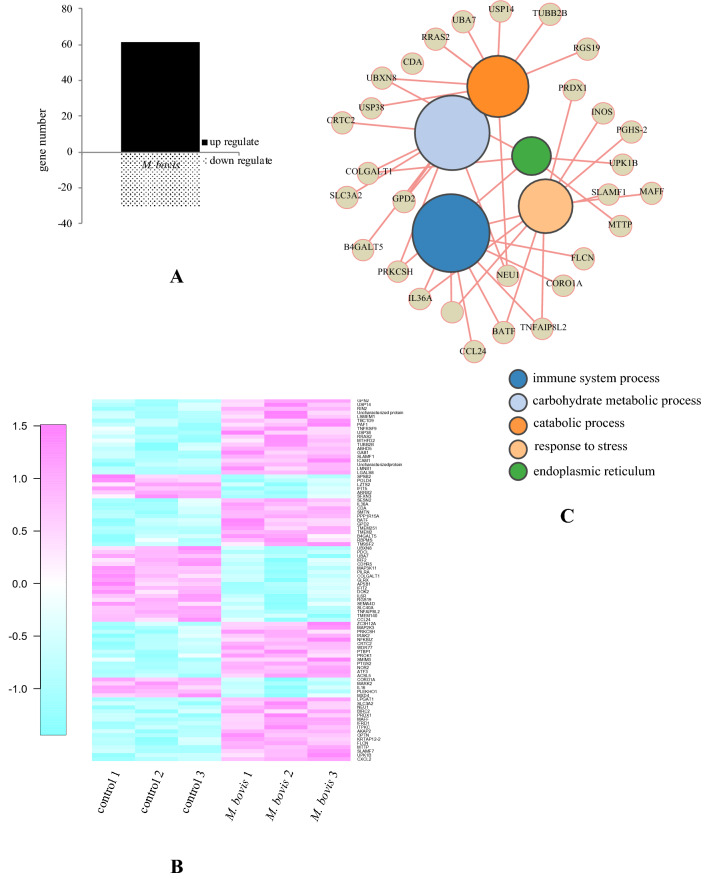
**Microarray analysis in neutrophils stimulated with** ***M. bovis***. Bovine neutrophils were evaluated at 3 h after stimulation with *M. bovis* in three cows. **A** Number of significantly (*t*-tests, *P* < 0.025 and ≥ twofold change) downregulated or upregulated mRNA. **B** Heat map analysis of genes with significantly (*t*-tests, *P* < 0.025 and ≥ twofold change) different gene expression levels by microarray analysis in neutrophils stimulated with *M. bovis*. **C** Gene ontology enrichment analysis of genes with significantly (*t*-tests, *P* < 0.025 and ≥ twofold change) different gene expression levels by microarray analysis in neutrophils stimulated with *M. bovis*.

**Figure 2:**
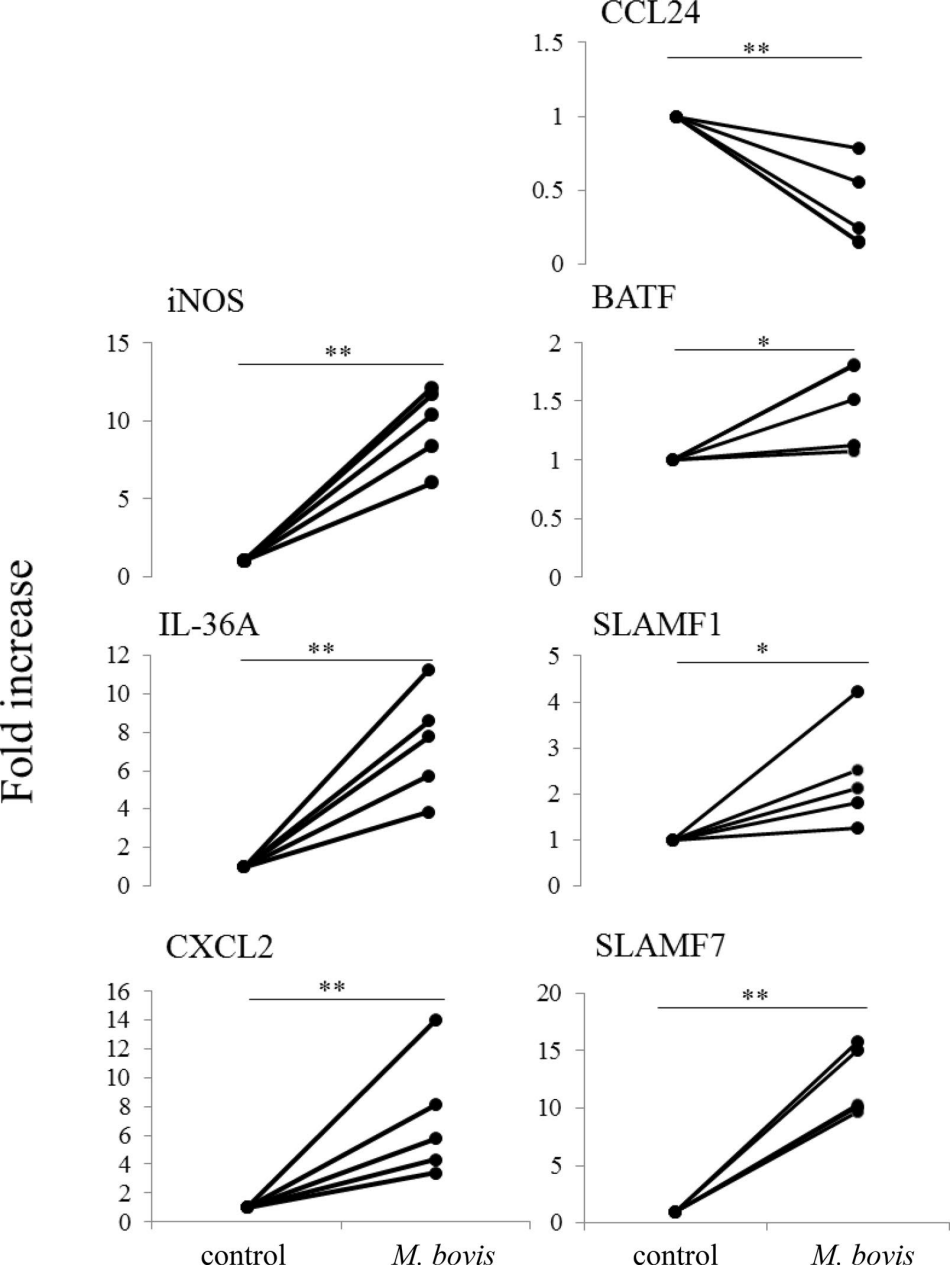
**Validation for the microarray analysis of mRNA expression in neutrophils stimulated with** ***M. bovis***. Bovine neutrophils were evaluated at 3 h after stimulation with *M. bovis* in three cows. Validation of mRNA expression for immune response-related factors confirmed by microarray analysis; significant difference at *P < 0.05 or **P < 0.01 compared to unstimulated controls.

We investigated gene expression in neutrophils stimulated with live *M. bovis* using an Agilent Bovine Gene Expression Microarray. Statistical analysis revealed that 61 genes in neutrophils stimulated with live *M. bovis* were significantly increased and 30 significantly decreased (*P* < 0.025 with > twofold increase) compared to unstimulated neutrophils (Figure 1A and Additional file 3). Expression gene patterns with significant differences were visualized using a heat map (Figure 1B). The *M. bovis* stimulated and unstimulated groups showed similar expression patterns. The gene set related to function did not significantly recognize change, but that of the immune system and carbohydrate metabolic processes were increased (Figure 1C and Additional file 2). To validate these results, genes related to the immune system were quantified using real-time PCR (Figure 2). Inducible NO synthase (iNOS), interleukin 36A (IL-36A), chemokine C-X-C motif ligand 2 (CXCL2), and signaling lymphocytic activation molecule (SLAM) family member 7 (SLAMF7) mRNA expression in neutrophils after 3 h of stimulation with *M. bovis* were significantly (*P* < 0.01) increased compared to unstimulated controls. Basic leucine zipper transcription factor ATF-like (BATF) and SLAM family member 1 (SLAMF1) mRNA expression also were significantly increased (*P* < 0.05). C–C motif chemokine ligand 24 (CCL24) was significantly decreased (*P* < 0.01) compared to unstimulated controls.

### Quantification of proinflammatory cytokine mRNA expression

**Figure 3:**
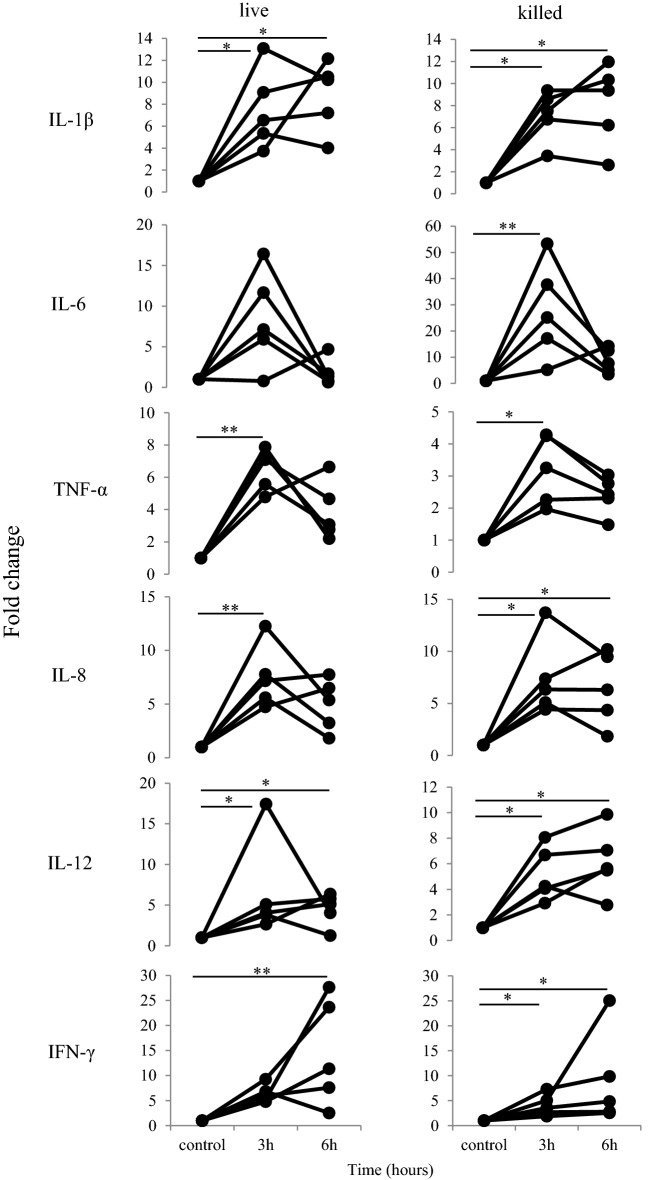
**mRNA expression of proinflammatory cytokine related genes after stimulation with** ***M. bovis***. Bovine neutrophils were evaluated at 3 or 6 h after stimulation with live or heat-killed *M. bovis* in five cows. The mRNA expression of IL-1β, IL-6, TNF-α, IL-8, IL-12, and IFN-γ was determined by qPCR and expressed as a fold increase, as described in the Materials and methods. The data were expressed in five cows; significant difference at *P < 0.05 or **P < 0.01 compared to unstimulated controls.

Expression of proinflammatory cytokine, IL-1β, IL-6, tumor necrosis factor α (TNF-α), IL-8, IL-12, and interferon γ (IFN-γ) mRNA in neutrophils stimulated with live or heat-killed *M. bovis* at 3 and 6 h was evaluated by qPCR (Figure 3). These cytokines showing mRNA expression in neutrophils stimulated with live *M. bovis* for 3 h were significantly (IL-1β and IL-12, *P* < 0.05; TNF-α and IL-8, *P* < 0.01) increased compared to unstimulated cells as were those stimulated with heat-killed *M. bovis* (IL-1β, TNF-α, IL-8, IL-12, and IFN-γ, *P* < 0.05; IL-6, *P* < 0.01). After 6 h of stimulation with *M. bovis*, IL-1β (live and heat-killed, *P* < 0.05), IL-8 (heat-killed, *P* < 0.05), IL-12 (live and heat-killed, *P* < 0.05), and IFN-γ (live, *P* < 0.01, and heat-killed, *P* < 0.05) demonstrated significantly increased mRNA expression in neutrophils.

### Evaluation producing NO and intracellular ROS of neutrophils stimulated with *M. bovis*

**Figure 4:**
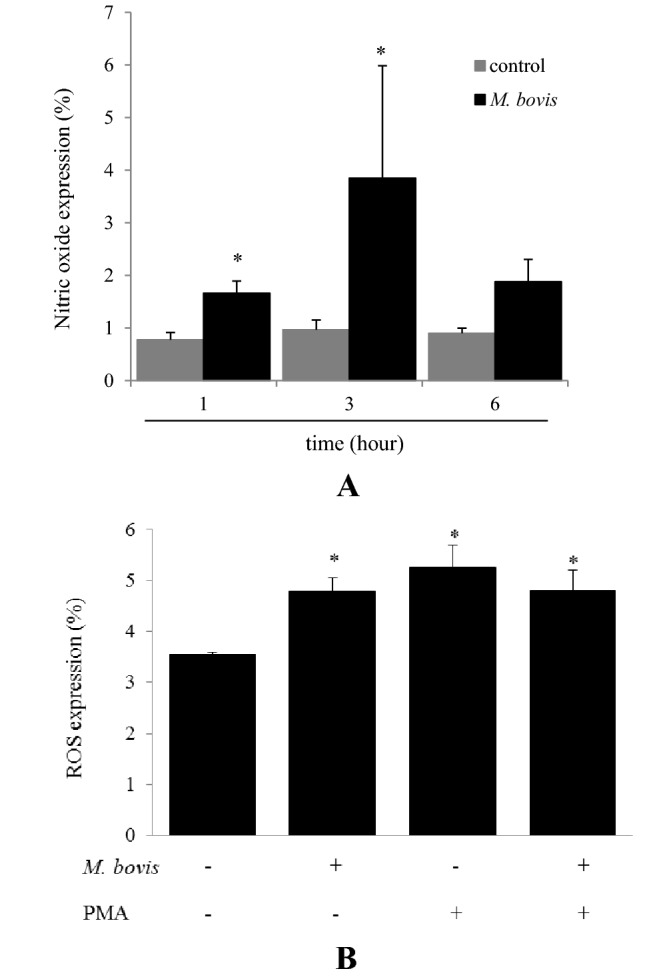
**Quantity of NO and ROS production in neutrophils stimulated with** ***M. bovis***. **A** Bovine neutrophils were evaluated at 1, 3, and 6 h after stimulation with *M. bovis* in five cows. The ratio of NO production cells is shown. Data were expressed as means ± SE in five cows; significant difference at *P < 0.05 compared to unstimulated controls. **B** Neutrophils were incubated with *M. bovis* (MOI of 1000) and/or PMA for 30 min. The ratio of ROS production cells is shown. Data were expressed as means ± SE of five cows; significant difference at *P < 0.05 compared to unstimulated controls.

NO production of neutrophils stimulated with *M. bovis* was significantly increased (*P* < 0.05) at 1 and 3 h compared to controls (Figure 4A). Intracellular ROS production of neutrophils stimulated with *M. bovis*, *M. bovis* and PMA, or PMA was significantly increased (*P* < 0.05) compared to unstimulated controls (Figure 4B).

### Evaluation of the apoptotic, nonapoptotic, dead, or live cells stimulated with *M. bovis*

**Figure 5:**
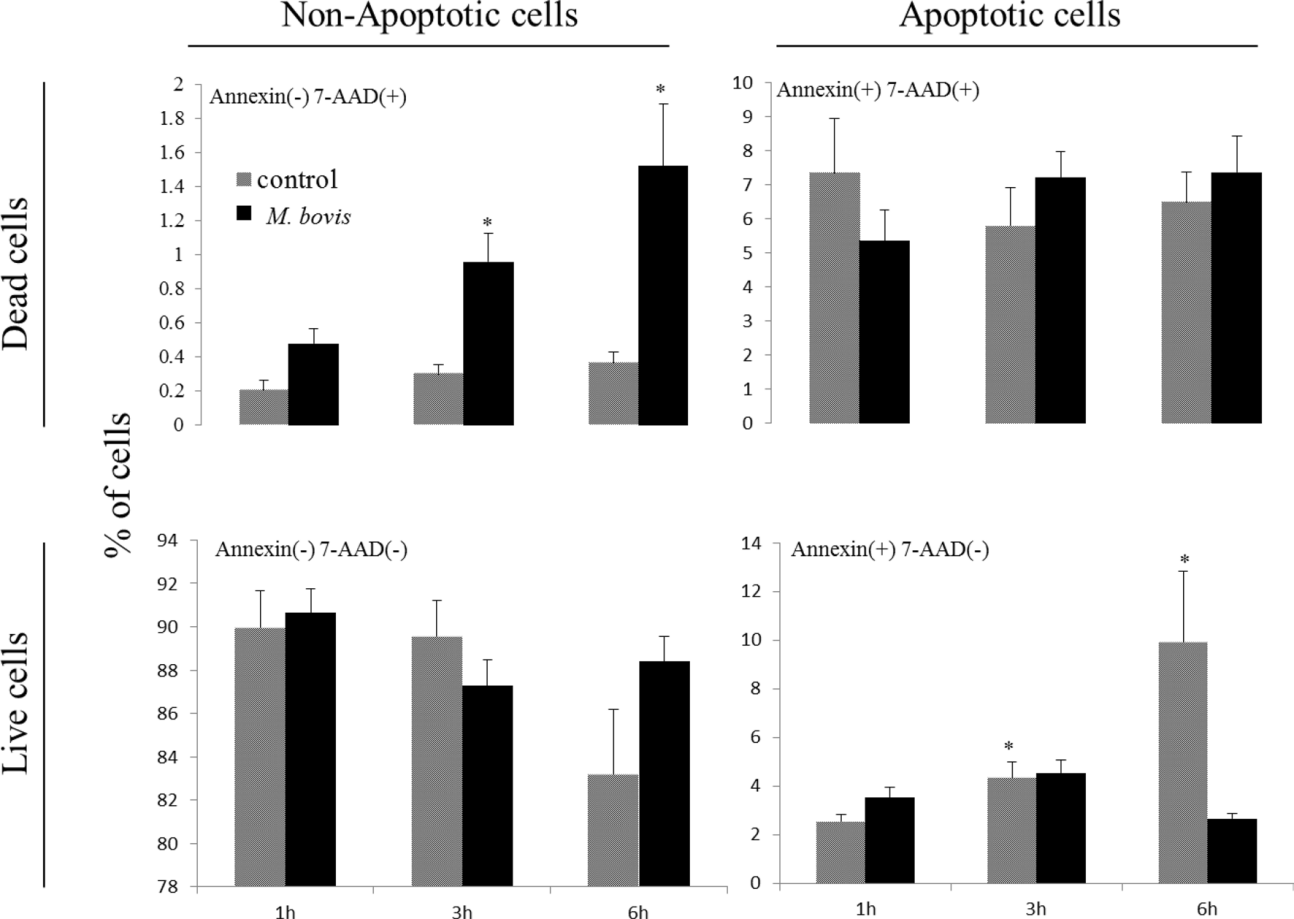
**Detection of apoptotic cells in neutrophils stimulated with** ***M. bovis***. Bovine neutrophils were evaluated at 1, 3, and 6 h after stimulation with *M. bovis* in five cows. The ratio of positive or negative neutrophils in Annexin V and/or 7-AAD is shown. Annexin V-positive/negative cells were defined as apoptotic/nonapoptotic cells, and 7-AAD–positive/negative cells were defined as dead/live cells. Data were expressed as means ± SE in five cows; significant difference at *P < 0.05 compared to unstimulated controls.

The ratio of apoptotic cells in neutrophils stimulated with *M. bovis* at an MOI of 1000 for 1, 3, and 6 h is shown Figure 5. The ratios of Annexin-positive and 7-AAD–negative cells (early apoptosis) in bovine neutrophils was significantly increased (*P* < 0.05) at 3 (4.36%) and 6 (9.92%) h compared to that at 1 h (2.54%). The ratios of Annexin-negative and 7-AAD–positive cells (dead cells other than nonapoptotic cells) in bovine neutrophils stimulated with *M. bovis* were significantly increased (*P* < 0.05) at 3 (0.96%) and 6 (1.52%) h compared to that at 1 h (0.20%). The ratios of Annexin-negative and 7-AAD–negative cells (live cells) in nonstimulated cells tended to decrease in a time-dependent manner, and those of Annexin-positive and 7-AAD–positive cells (late apoptotic and necrotic cells) in neutrophils stimulated with *M. bovis* tended to increase in a time-dependent manner.

### Observation of NETs formation

**Figure 6:**
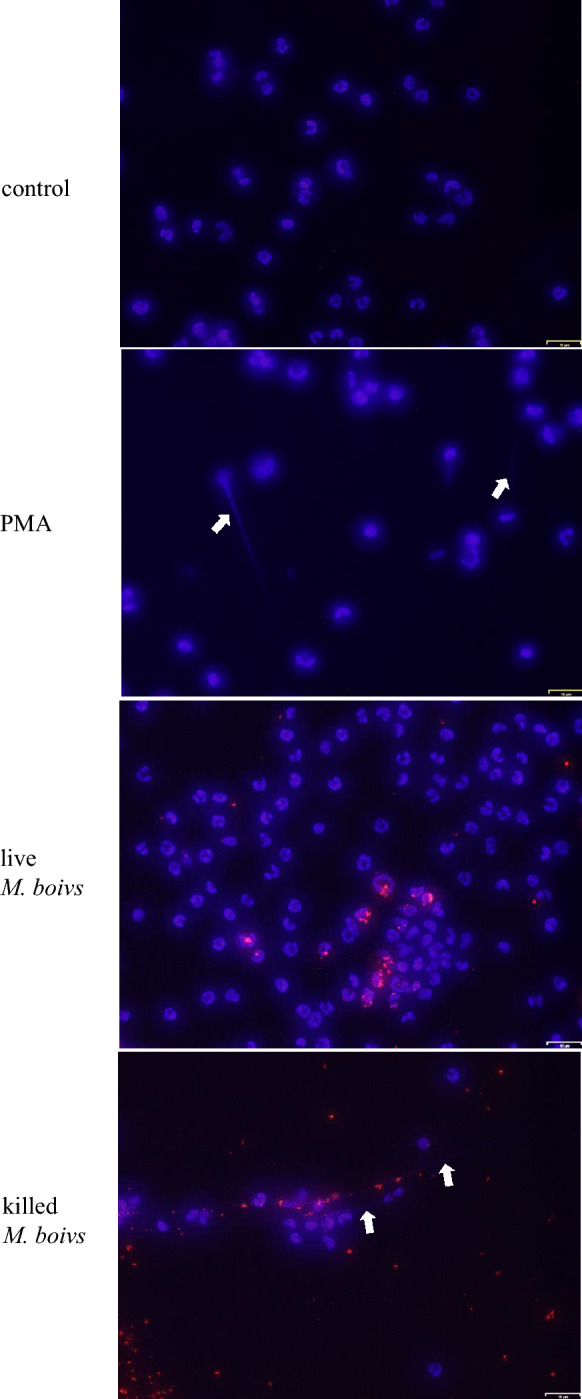
**Observation of NETs formation**. Bovine neutrophils were evaluated at 30 min after stimulation with live or heat-killed *M. bovis* (MOI of 100)/PMA in three cows (unstimulated controls, PMA-stimulated, live *M. bovis*-stimulated, and heat-killed *M. bovis*-stimulated). DNA was stained using DAPI, and *M. bovis* was labeled with rhodamine. The white arrow indicates NETs formation. The white bar indicates 16 μm, and representative micrographs are shown.

NETs formation in neutrophils stimulated with live or heat-killed *M. bovis* is shown in Figure 6. NETs formation was detected in neutrophils stimulated with PMA as an inducer of NETs formation, whereas it was not observed in neutrophils stimulated with live *M. bovis* and unstimulated controls. However, in neutrophils stimulated with heat-killed *M. bovis*, NETs formation was recognized, and *M. bovis* was localized to be on NETs.

## Discussion

We studied the innate immunity of bovine neutrophils to *M. bovis*, and we especially reported a transcriptome analysis of bovine neutrophils stimulated with *M. bovis* and its immune-related functional analysis. Proinflammatory cytokines, such as iNOS, IL-36A, and CXCL2 mRNA, were increased in neutrophils stimulated with *M. bovis* as determined in the transcriptome analysis using microarray and qPCR for validation. iNOS induced NO production with antibacterial activity and was related to the formation of NETs [11]. Previous studies [14] did not show the production of neutrophil NO upon *M. bovis* stimulation, which may be due to the use of different MOIs and detection methods. CXCL2 is a chemokine related to priming of neutrophils [21] and enhanced antibacterial activity [22]. SLAMF1 and SLAMF7 affect the development of T cells [23]. BATF was reported to regulate T and B lymphocytes in immune response and differentiation [24]. *IL-1β*, *IL-6*, and *TNF-α* are known rapid transcription-genes, proinflammatory cytokines, and the peak time point in these genes was mainly 3 h. IL-1β- and IL-8-related migration and activation of neutrophils reportedly had an important role in maintaining inflammation in *M. bovis* infectious diseases. IL-12 and IFN-γ mainly reached peak levels at 6 h after stimulation with *M. bovis*, which may be due to the activated neutrophils and a type 1 immune response [25]. The production of IL-12 and TNF-α by *M. bovis*-stimulated neutrophils has been also observed in a previous study [14], which is in line with our present results. In gene ontology enrichment analysis, most dynamically changed genes indicated an immune response, and *M. bovis* induced an innate immune response in bovine neutrophils. The carbohydrate metabolic process was the second largest gene set changed, and it includes the production of activated oxygen. Activated oxygen production in neutrophils stimulated with *M. bovis* was not recognized as reported previously [26, 27]. However, this study demonstrated that intracellular production of activated oxygen was recognized. Mycoplasma species were known to have a resistance factor for activated oxygen [28, 29], which suggested that, although neutrophils stimulated with *M. bovis* produced activated oxygen, *M. bovis* attenuated extracellular activated oxygen. Because ROS were required for NETs formation [9], *M. bovis* may have potential in inducing NETs formation. However, as ROS-independent NETs formation has been reported recently [30, 31], further studies are needed on *M. bovis*-induced ROS production. The production of NO in neutrophils reportedly contributed to NETs formation [11]. In our study, neutrophils stimulated with *M. bovis* in transcriptome analysis and qPCR analysis showed increased iNOS mRNA expression, and NO production was increased in neutrophils stimulated with *M. bovis*. It had been reported that iNOS was strongly expressed in lungs of calves with coagulative and caseous necrosis lesion after infection with *M. bovis* [10]. Thus, it was suggested that the immune response of neutrophils was involved in the pathogenesis in mycoplasma infectious disease. Neutrophils stimulated with *M. bovis* showed an increased ratio of nonapoptotic cell death compared to unstimulated controls. It was considered that NETosis, which is cell death following NETs formation, contributed to nonapoptotic cell death. Mulongo et al. [32] and Maina et al. [33] reported that *M. bovis* delayed the apoptosis of monocytes and macrophages, respectively. Instead of suppressing apoptosis in neutrophils, *M. bovis* may promote NETs formation. In the study by Jimbo et al., the production of elastase, which is used as an index of NETs, was observed from neutrophils under stimulation with live *M. bovis* [14]. NETs formation was not observed in live bacteria stimulation, but it was observed in heat-killed bacteria stimulation. This result cannot immediately indicate that *M. bovis* has not induced NETs formation. This suggested that NETs were degraded by *M. bovis* nuclease in live bacteria as reported previously [26, 27], whereas in heat-killed *M. bovis*, the nuclease was inactivated by heat treatment and thus NETs formation was observed. The increased expression of proinflammatory cytokine mRNA even after stimulation with heat-killed bacteria suggested that *M. bovis* has the potential to induce NETs formation even in killed bacteria.

In conclusion, we demonstrated the innate immune response gene expression of bovine neutrophils stimulated with *M. bovis*, its related function expression related to NETs formation, and interaction between *M. bovis* and bovine neutrophils.

## Supplementary Information

**Additional file 1. Sequences of oligonucleotide primers.**

**Additional file 2. The biological process GO term enrichment in bovine neutrophils stimulated with *M. bovis***. “Corrected *P*-Value” is correction for multiple testing.

**Additional file 3. Transcriptome analysis in neutrophils stimulated with *M. bovis***. Bovine neutrophils were evaluated by microarray analysis at 3 h after stimulation with *M. bovis* in three cows. After normalization and gene annotation, fluorescence intensity in significantly (*t*-tests, *P* < 0.025 and a ≥ twofold change) upregulated or downregulated genes was shown for each cow (control as unstimulated control vs. Mb as *M. bovis* stimulation).

## Data Availability

All datasets are presented in the paper or additional files supporting the manuscript.

## Abbreviations

*M. bovis*: *Mycoplasma bovis*
*iNOS*: Inducible nitric oxide
NETs: Neutrophil extracellular traps
ROS: Reactive oxygen species
PPLO: Pleuropneumonia-like organisms
PBS: Phosphate-buffered saline
CFU/mL: Colony-forming units per milliliter
FBS: Fetal bovine serum
MOI: Multiplicity of infection
TRNA: Total RNA
*GAPDH*: Glyceraldehyde-3-phosphate dehydrogenase
*YWHAZ*: Tryptophan 5-monooxygenase activation protein zeta polypeptide
DAPI: 4,6-Diamidino-2-phenylindole, dilactate
IL-36A: Interleukin 36A
CXCL2: Chemokine C-X-C motif ligand 2
SLAMF7: Signaling lymphocytic activation molecule family member 7
BATF: Basic leucine zipper transcription factor ATF-like
SLAMF1: Signaling lymphocytic activation molecule family member 1
CCL24: C–C motif chemokine ligand 24
TNF-α: Tumor necrosis factor α
IFN-γ: Interferon γ
