# Supplementary material for: Innate immune response in bovine neutrophils stimulated with Mycoplasma bovis
Source: Vet Res. 2021 Apr 16;52:58. doi: 10.1186/s13567-021-00920-2 (PMC8052696; doi:10.1186/s13567-021-00920-2)
Supplement: Supplementary file 1 — Additional file 1. Sequences of oligonucleotide primers. [file 13567_2021_920_MOESM1_ESM.docx]

**Additional file 1 Sequences of oligonucleotide primers.**

| **Gene name** | **Primer sequence (5′-3′)** | |  |  | |  |
| --- | --- | --- | --- | --- | --- | --- |
| β-actin | F: AGC AAG CAG GAG TAC GAT GAG | | NM_173979 | [19] | |  |
|  | R: ATC CAA CCG ACT GCT GTC A | |  |  | |  |
| YWHAZ | F: GCA TCC CAC AGA CTA TTT CC | | GU817014 | [20] | |  |
|  | R: GCA AAG ACA ATG ACA GAC CA | |  |  | |  |
| GAPDH | F: GGC GTG AAC CAC GAG AAG TAT AA | | NM_001034034 | [19] | |  |
|  | R: CCC TCC ACG ATG CCA AAG T | |  |  | |  |
| IL-1β | F：AGT GCC TAC GCA CAT GTC TTC | | NM 174093 | [12] | |  |
|  | R: TGC GTC ACA CAG AAA CTC GTC | |  |  | |  |
| IL-6 | F: ATC AGA ACA CTG ATC CAG ATC C | | NM 173923 | [12] | |  |
|  | R: CAA GGT TTC TCA GGA TGA GG | |  |  | |  |
| IL-8 | F: GAA GAG AGC TGA GAA GCA AGA TCC | | NM 173925 | [12] | |  |
|  | R: ACC CAC ACA GAA CAT GAG GC | |  |  | |  |
| IL-12p40 | F: CAT CAG GGA CAT CAT CAA AC | | NM 174356 | [12] | |  |
|  | R: AAC GTC AGG GAG AAG TAG GA | |  |  | |  |
| IFN-γ | F: TCA AAT TCC GGT GGA TGA TCT GC | | NM_174086 | [12] | |  |
|  | R: GAC CAT TAC GTT GAT GCT CTC CG | |  |  | |  |
| TNF-α | F: TCT TCT CAA GCC TCA AGT AAC AAG C | | NM 173966.3 | [12] | |  |
|  | R: CCA TGA GGG CAT TGG CAT AC | |  |  | |  |
| SLAMF1 | F: AGT CTG GAC CTT CAG GCA AC | | NM_174184 |  | |  |
|  | R: GTA CAG GCA GCC AAG GTG TA | |  |  | |  |
| SLAMF7 | F: TCC TGA AGA GAT GCC CGA GT | | NM_001191358 |  | |  |
|  | R: ATC TGA CAA CAT GGG CAG GG | |  |  | |  |
| CCL24 | F: CCA GGC AGG AGT GAT CTT CA | | NM_001046596 |  | |  |
|  | R: CCC TAG CGG AGG CTT TCT TC | |  |  | |  |
| BATF | F: CCC AGT GAT GGG TCA AGC AT | | NM_001206278 |  | |  |
|  | R: AGC CCT TGC CAG ATT GGT TT | |  |  | |  |
| IL-36A | F: GTG GAG GCT GTC CTG TGA TT | | XM_005192441 |  | |  |
|  | R: AGC AGA GAA CAA CCC TCA TCC | |  |  | |  |
| iNOS | F: CTT GAT TGC ACC GCT TGG AG | | NM_001076799 |  | |  |
|  | R: CAA GAG GCA GAC TGG GGT TT | |  |  | |  |
| CXCL2 | F: TCA GGA AGT GTG TCT CAA CCC | | NM_174299 |  | |  |
|  | R: TTC TGT AGG GGC AGG GTC TA | |  |  | |  |
| F, forward; R, reverse; bps, base pairs | |  | | |  | |
