# Supplementary material for: Innate immune response in bovine neutrophils stimulated with Mycoplasma bovis
Source: Vet Res. 2021 Apr 16;52:58. doi: 10.1186/s13567-021-00920-2 (PMC8052696; doi:10.1186/s13567-021-00920-2)
Supplement: Supplementary file 2 — Additional file 2. The biological process GO term enrichment in bovine neutrophils stimulated with M. bovis. “Corrected P-Value” is correction for multiple testing. [file 13567_2021_920_MOESM2_ESM.docx]

**Additional file 2 GO analysis in Neutrophils stimulated with *M. bovis***

| Description | *P*-Value | Corrected *P*-Value |
| --- | --- | --- |
| immune system process | 5.68E-04 | 0.0806246 |
| carbohydrate metabolic process | 4.49E-03 | 0.637681 |
| catabolic process | 0.0152978 | 1 |
| response to stress | 0.0212556 | 1 |
| endoplasmic reticulum | 0.0342777 | 1 |
| cytoplasm | 0.0359709 | 1 |
| locomotion | 0.0372123 | 1 |
| embryo development | 0.0428116 | 1 |
